# The Latin American Spanish Preclinical Alzheimer's Cognitive Composite (LAS‐PACC5): Adapted for Spanish‐Speaking Latino Populations

**DOI:** 10.1002/alz70857_105553

**Published:** 2025-12-25

**Authors:** Nadeshka J Ramirez‐Perez, Lusiana Martinez, Randy Medrano, Nikole A Bonillas Félix, Alexandre Closs Fanfa Ribas, Liliana Ramirez Gomez, Averi Giudicessi, Jairo E. Martinez, Jorge Alcina, Isabel Solis, Marta Gonzalez Catalan, Clara Vila‐Castelar, Daniel G Saldana, Yakeel T. Quiroz

**Affiliations:** ^1^ 95 Grant Avenue, Medford, MA, USA; ^2^ Massachusetts General Hospital, Harvard Medical School, Boston, MA, USA; ^3^ Massachusetts General Hospital, Boston, MA, USA; ^4^ Boston University, Boston, MA, USA

## Abstract

**Background:**

Validated cognitive assessments in Spanish are critical for evaluating Latino populations at risk for Alzheimer's disease (AD). To address this need, we developed a Latin American Spanish version of the Preclinical Alzheimer's Cognitive Composite (LAS‐PACC5), a specialized tool designed to detect early cognitive changes in individuals at elevated risk for AD. Using a sample of cognitively unimpaired Latino individuals, we examined the associations between LAS‐PACC5 and existing cognitive measures. We then assessed its ability to differentiate between unimpaired and impaired individuals.

**Method:**

A total of 191 Spanish‐speaking participants (139 cognitively unimpaired, 52 impaired) from the Boston Latino Aging Study (BLAST) were included (MAge = 67.98 ± 8.246 years; MEducation = 10.90 ±4.974 years; 74.41% female). They completed the adapted LAS‐PACC5, replacing two subtests (WAIS Digit Symbol Substitution with oral Symbol‐Digit Modalities Test, and WMS‐R Logical Memory with NEUROPSI Logical Memory Delayed Recall), while retaining the Mini‐Mental State Examination (MMSE), the Free and Cued Selective Reminding Test (FCSRT), and Category Fluency (Animals). Additional cognitive tests (NEUROPSI Word List, semantic fluency: Fruits, and the Multilingual Naming Test‐MINT) were used for comparison. All assessments were administered in Spanish, and the LAS‐PACC5 was computed as a summary of z‐scores from the five core measures, using the mean and SD of the unimpaired group as the reference scores. Group comparisons and associations were analyzed using Mann‐Whitney U test and partial Spearman correlations, adjusting for age and education.

**Result:**

The LAS‐PACC5 showed positive correlations with the NEUROPSI Word List (rs = 0.32, p < .001), semantic fluency (fruits) (rs=0.46, *p* < .001), and the MINT (*r* = 0.34, p < .001). Additionally, LAS‐PACC5 distinguished between unimpaired participants (M = ‐0.19 ± 3.34) and impaired participants (M = ‐13.38 ± 11.40) with a significant difference in the distribution of summary scores (*p* < .001) and a large effect size (*r* =  ‐0.66).

**Conclusion:**

The LAS‐PACC5 showed convergent validity with memory and language measures and successfully distinguished between Latino individuals with and without cognitive impairment, supporting its reliability for assessing overall cognition and detecting cognitive impairment in Spanish‐speaking older Latinos. Future research should include longitudinal follow‐up, larger samples, and explore associations with AD‐related biomarkers, including imaging and blood‐based measures.

